# Autoantibody repertoire characterization provides insight into the pathogenesis of monogenic and polygenic autoimmune diseases

**DOI:** 10.3389/fimmu.2023.1106537

**Published:** 2023-02-10

**Authors:** Thomas Clarke, Pan Du, Satyendra Kumar, Shinji L. Okitsu, Mark Schuette, Qi An, Jinyang Zhang, Evgeni Tzvetkov, Mark A. Jensen, Timothy B. Niewold, Elise M. N. Ferre, Julie Nardone, Michail S. Lionakis, Jaromir Vlach, Julie DeMartino, Andrew T. Bender

**Affiliations:** ^1^ TIP Immunology, EMD Serono, Billerica, MA, United States; ^2^ Protein Engineering and Antibody Technologies, Merck KGaA, Darmstadt, Germany; ^3^ Department of Immunology, Division of Rheumatology, Mayo Clinic, Rochester, MN, United States; ^4^ Fungal Pathogenesis Section, Laboratory of Clinical Immunology and Microbiology (LCIM), National Institute of Allergy and Infectious Diseases (NIAID), Bethesda, MD, United States

**Keywords:** autoantibody, autoimmune disease (AD), autoimmune polyendocrinopathy candidiasis ecotodermal dystrophy (APECED), B cell receptor (BCR), Sjogren's syndrome, systemic lupus erythematosus (SLE)

## Abstract

Autoimmune diseases vary in the magnitude and diversity of autoantibody profiles, and these differences may be a consequence of different types of breaks in tolerance. Here, we compared the disparate autoimmune diseases autoimmune polyendocrinopathy–candidiasis–ecto-dermal dystrophy (APECED), systemic lupus erythematosus (SLE), and Sjogren’s syndrome (SjS) to gain insight into the etiology of breaks in tolerance triggering autoimmunity. APECED was chosen as a prototypical monogenic disease with organ-specific pathology while SjS and SLE represent polygenic autoimmunity with focal or systemic disease. Using protein microarrays for autoantibody profiling, we found that APECED patients develop a focused but highly reactive set of shared mostly anti-cytokine antibodies, while SLE patients develop broad and less expanded autoantibody repertoires against mostly intracellular autoantigens. SjS patients had few autoantibody specificities with the highest shared reactivities observed against Ro-52 and La. RNA-seq B-cell receptor analysis revealed that APECED samples have fewer, but highly expanded, clonotypes compared with SLE samples containing a diverse, but less clonally expanded, B-cell receptor repertoire. Based on these data, we propose a model whereby the presence of autoreactive T-cells in APECED allows T-dependent B-cell responses against autoantigens, while SLE is driven by breaks in peripheral B-cell tolerance and extrafollicular B-cell activation. These results highlight differences in the autoimmunity observed in several monogenic and polygenic disorders and may be generalizable to other autoimmune diseases.

## Introduction

The natural process of immunity is tightly controlled with multiple levels of regulation to safeguard against the development of reactivity to self. However, breaks in tolerance do occur for various reasons, often with devastating consequences as autoimmune diseases can greatly decrease the quality of life for sufferers and may be fatal. Understanding how breaks in tolerance can occur and identifying which pathways to stimulate or inhibit to restore tolerance may help identify next-generation treatment strategies for autoimmune diseases.

A number of monogenic autoimmune diseases have been identified wherein a single mutation triggering impaired tolerance leads to clinical disease. One such monogenic autoimmune disease is autoimmune polyendocrinopathy–candidiasis–ectodermal dystrophy APECED, also known as autoimmune polyglandular syndrome type-1 (APS-1), which is caused by mutations in the gene *AIRE* (1–3). *AIRE* is expressed in medullary thymic epithelial cells (mTEC) and plays a major role in the negative selection of autoreactive T-cells and maintenance of central tolerance. APECED patients experience severe autoimmunity against a wide variety of tissues and organs, with the most common being endocrine organs (1). Ironically, autoimmunity against cytokines also leads to immunodeficiency that contributes to susceptibility to severe SARS-CoV2 and mucosal *Candida* infections (4–7).

Autoimmunity may also have a polygenic etiology and arises due to a combination of factors including environmental triggers and often involves a break in peripheral tolerance. Diseases of this nature include systemic lupus erythematosus (SLE), Sjogren’s syndrome (SjS), multiple sclerosis, and rheumatoid arthritis (8, 9). These diseases vary in the breadth of autoreactivity, and the number of tissues and organs involved. For example, SLE is a disease with very broad autoreactivity and involvement of multiple organ systems (10), while SjS is a disease with pathology mainly confined to glands producing saliva and tears and reactivity against a limited number of antigens, most prominently Ro and La (11).

The autoreactivity profile of APECED patients has been studied recently and new insights have been gained as to the antigens targeted and the breadth of their B-cell receptor (BCR) repertoire. Broad profiling of APECED patients has established a list of common reactivities among these patients and identified anti-cytokine antibodies targeting type I interferons, IL-22, and IL-17 (12–17). The mechanism by which autoimmunity arises with the loss of *AIRE* has been studied and links loss of T-cell tolerance to an accumulation of autoreactive B-cells (15). However, much is still not understood about how and why the severe autoimmunity develops in APECED as *AIRE* knockout mice on the C57BL/6 background have a less severe phenotype than disease seen in patients (18). A loss in Tregs (15) or other environmental factors may also contribute to autoimmunity and disease severity. Recent research has identified aberrant type 1 responses to be a critical pathogenic mechanism that leads to mucosal fungal infections in APECED patients (19).

Polygenic autoimmune diseases such as SLE and SjS have different etiologies compared to APECED and, consequently, differences in their reactivity profile. SLE may be considered the extreme case for loss of tolerance with many SLE patients showing positivity for a wide range of autoantibodies and suffering from a range of clinical manifestations (20). A recent report comparing the BCR repertoire of immune-mediated diseases, including SLE, illustrated both the considerable clonal diversity and expansion observed in SLE patients relative to the other diseases studied (21). The conventional understanding of SLE pathogenesis and breaks in tolerance involves dysregulated apoptosis or necrosis-releasing cellular debris that is not cleared and activates autoreactive B-cells, often with danger signals (such as Toll-like receptor [TLR] ligands) serving to drive activation and expansion of the autoreactive B-cells which might otherwise not be responsive (22). Recent work highlighted a population of CD27 and IgD double-negative B-cells that are hyper-responsive to TLR7 signaling and expanded in lupus patients (23). The autoantibodies from activated cells can form immune complexes with the circulating autoantigens to further activate immune cells perpetuating the cycle. This process of loss of tolerance in the periphery without T-cell help may underlie the differences in BCR repertoire between diseases.

Here, we have aimed to compare the autoantibody profiles and B-cell repertoires of SLE, SjS, and APECED patients to gain insight into the etiology of breaks in tolerance that lead to autoimmunity. We have used both antibody profiling and BCR sequence analysis as two different methods to characterize B-cell autoimmunity. Directly contrasting these three diseases is a novel approach to learn more about autoimmunity and our results can be used to build a model of pathogenesis that brings together different immunological concepts already known about these diseases.

## Materials and methods

### Human subjects

To study the B-cell repertoire of patients with autoimmune conditions, blood was collected from patients with APECED (n=20), SjS (n=26), SLE (n=43), and healthy controls (HC) (n=18). Patients aged 18–70 years with either APECED, SLE or SjS were enrolled. SLE was defined as meeting at least four ACR formal diagnostic criteria (24) and anti-dsDNA positive. A SjS diagnosis included meeting at least four of the American–European consensus group classification for primary SjS criteria (25), including either histopathology or autoantibodies, as well as a previously established diagnosis of SjS and no other autoimmune connective tissue disease, positive dry eye and dry mouth symptoms, and a positive test for anti-Ro/SSA and/or anti-La/SSB antibodies. APECED patients had to meet the clinical diagnostic criteria for APECED (presence of any 2 manifestations among the triad of mucocutaneous candidiasis, hypoparathyroidism, and adrenal insufficiency) and/or have confirmed biallelic mutations in *AIRE*. Blood samples were collected from patients with APECED at the NIH and HCs/SjS patients/SLE patients at the Mayo Clinic (Rochester, MN).

### ProtoArray analysis

IgG reactivity was profiled in plasma samples using the ProtoArray, which tests for reactivity against 9483 proteins immobilized on a glass slide. This approach has been used previously to identify reactivity profiles for patients with APECED (12–14) as well as SLE and SjS (26, 27). Patient samples were tested on the array and from the processed data Z scores were calculated versus the HC group. The spot intensities were processed using the PAA R script package (Bioconductor version: Release [3.15]) to handle background subtraction, batch filtering, cyclic loess normalization, and generation of data points for the volcano plots. For each spot, the intensities from the healthy controls were used as a baseline in calculating the Z-scores to identify enrichment or depletion of specific autoantigen reactivities. A Rosner test was used to identify outliers in the healthy controls that were excluded from the baseline (p<0.01, k=3). The mean and standard deviation of the baseline samples were used to calculate Z-scores across all samples for that spot. The Z-score matrix of the most variable spots was used to generate a heatmap using the pheatmap package.

### NGS gene expression analysis

Blood samples were collected into PAXgene RNA tubes (BD Biosciences). RNA was extracted and RNA-seq performed for gene expression analysis (Azenta Life Sciences, Chelmsford, MA). Strand-specific RNA-seq with rRNA and Globin Depletion was performed (~30M reads/sample). FASTQ files were processed and differential expression analysis was performed. The data has been uploaded to NCBI GEO under the accession number GSE222408.

### Isolation of switched memory B-cells

Switched memory B-cells (CD3-, CD19+, IgD-, IgM-) were isolated from peripheral blood and RNA was isolated and sequenced using IgG-specific primers. To compare the diversity and clonal expansion of the BCR repertoire in the different groups, a plot was constructed of the fraction of IgG UMIs for the 4000 top ranked clones for each subject.

### IgG amplification and NGS BCR library preparation

As the mechanism for the break in tolerance differs between the diseases and the amount of T-cell help is likely different, it is possible that affinity maturation could be altered and this may manifest in the distribution of VH gene family usage, CDR3 length, or SHM rate. These parameters were analyzed to try and gain more insight into the nature of the autoreactive B-cells in the patients. For each disease group, VH gene usage was determined for the 30 most highly used VH genes.

For full practical methods outlining how antibody profiling and BCR sequence analysis were performed, please see the Supplementary Material.

## Results

### ProtoArray profiling of APECED, SjS, and SLE patient autoantibody repertoires

**Figure 1:**
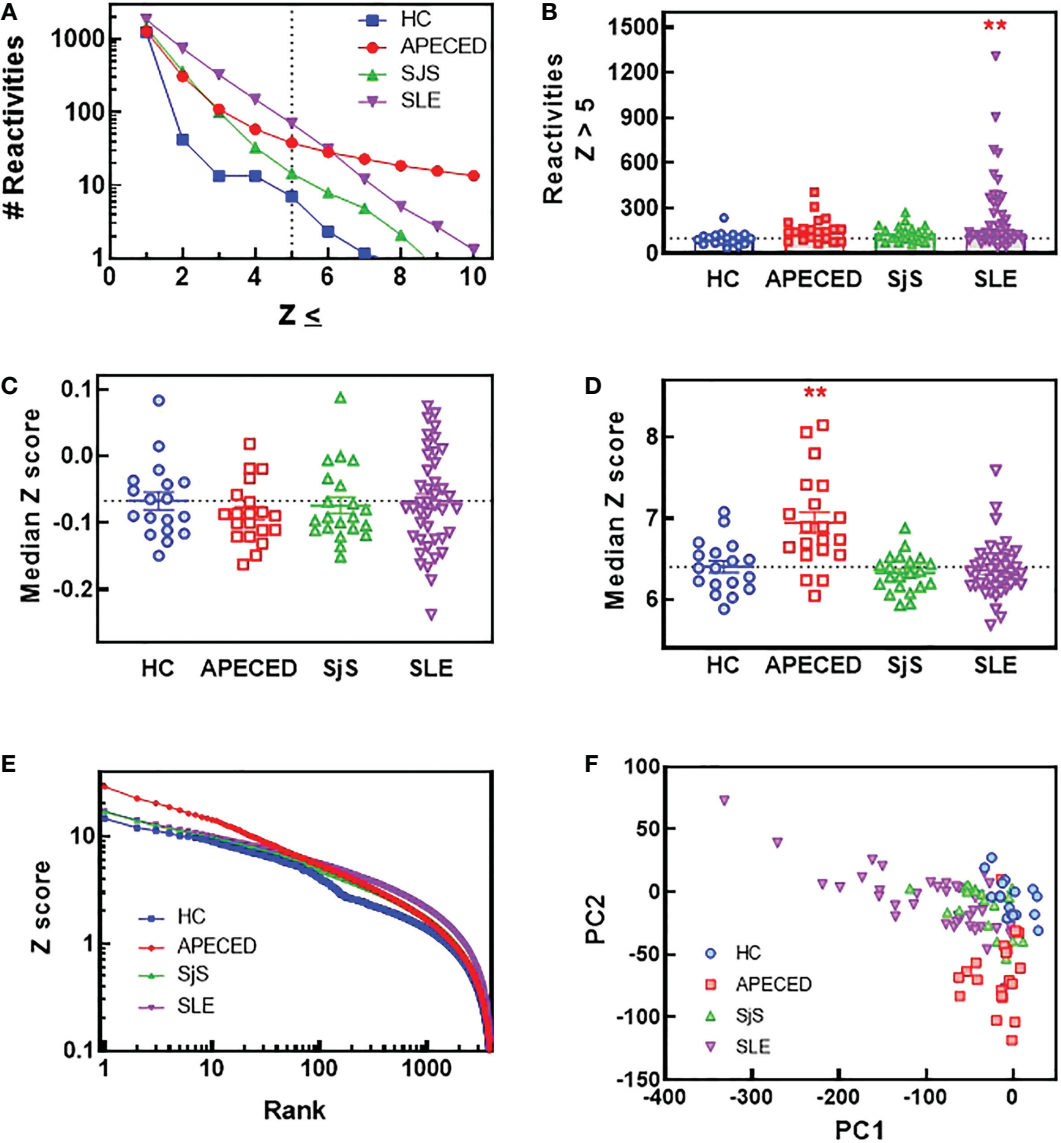
ProtoArray analysis of autoimmune patients reveals that autoantibody repertoires differ in the number of reactivities and their clonality between diseases. Plasma samples from patients and HCs were tested using the human ProtoArray covering 9483 proteins for IgG reactivity. Z scores were calculated to quantify reactivity for patients versus the HC group. **(A)** The median number of reactivities above a Z score threshold ranging from 1 to 10 were plotted for each group. **(B)** The number of reactivities with a median Z score >5 are plotted for each group. **(C)** The median Z score is plotted for each subject and also **(D)** for only reactivities with a Z score >5. **(E)** The top 4000 reactivities for each group are plotted ranked by highest Z score with the Z score for each reactivity on the Y axis. **(F)** A PCA plot was constructed of all subjects based on the ProtoArray data. (**significantly different from HC ANOVA p<0.01). HCs, healthy controls; IgG, immunoglobulin G; PCA, principal component analysis.

In order to compare the breadth and distribution of autoantibody reactivites in dissimilar autoimmune diseases, we screened plasma samples for IgG reactivity from APECED, SLE, and SjS patients and healthy controls (HC) against 9483 proteins on a single glass slide. Few reactivities were found in the HC group (**Figure 1A**) with a Z score >5, which was used as a cutoff for defining reactivities considered to be non-normal. When comparing patients with autoimmune conditions, it was noted that the SLE group had a higher number of reactivities at Z scores of <6, but at Z scores >6 the APECED group had the largest number of reactivities. When the number of reactivities with a Z score >5 was plotted for each subject (**Figure 1B**), the SLE group showed the largest inter-subject diversity with some individuals showing extremely high autoreactivity, and this diversity was also evident in a plot of the median Z scores (**Figure 1C**). However, when the median Z score was calculated for each subject using reactivities >5, then the median Z scores were significantly higher for patients with APECED (**Figure 1D**). Taken together, these observations suggest that patients with SLE have a large number of reactivities that are different from normal, but they may not be high affinity or present at high levels. In contrast, patients with APECED have a more limited number of autoreactivities but they are very high affinity and/or present at high concentrations. This difference between APECED and SLE is best illustrated by a plot of Z scores for each reactivity by rank (**Figure 1E**). The difference in the patient groups can also be seen in a principal component analysis plot of the ProtoArray data (**Figure 1F**) as there is an obvious separation of the SLE and APECED groups from the HCs. The SjS group has a very limited number of defined autoreactivities and is thus only slightly separated from the HC group.

**Figure 2:**
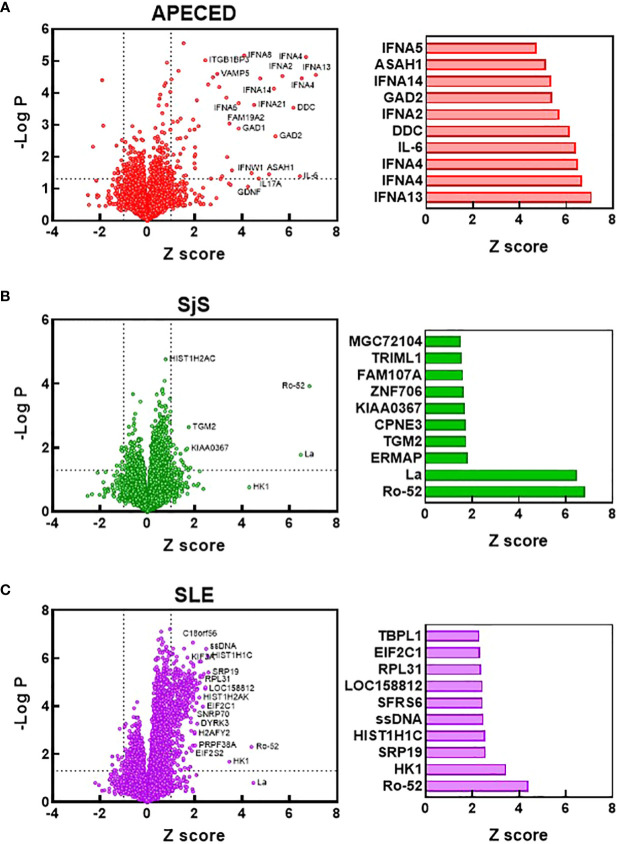
Identification of the most significantly elevated autoantibodies. Volcano plots were constructed based on the ProtoArray data for the APECED **(A)**, SjS **(B)**, and SLE **(C)** patient groups. Dotted lines define reactivities with a Z scores >1 and a p value of <0.05 (t test) compared with the HC group. Notable reactivities are labeled. Bar charts show the 10 reactivities that were significantly different from HCs (p<0.05) with the largest Z scores for each disease. APECED, autoimmune polyendocrinopathy–candidiasis–ecto-dermal dystrophy; SLE, systemic lupus erythematosus; SjS, Sjogren’s syndrome.

To compare the identities of the autoreactivities elevated in the different diseases from the ProtoArray analysis, volcano plots were constructed showing the Z scores and -log p values for autoreactivities increased in the different disease groups relative to the HC group (**Figures 2A–C**). Bar charts were also created to show the 10 reactivities for each disease that were significantly different from HCs (p<0.05) and had the highest Z score. The number of significantly elevated reactivities and the magnitude of increase differed greatly between the studied diseases. APECED samples showed a limited number of significantly increased reactivities against several antigens such as cytokines (in particular interferon alpha subtypes) and glutamic acid decarboxylase, which are characteristic of the disease (1) (**Figure 2A**). The SjS group had a very limited reactivity profile with reactivity almost exclusively against Ro-52 and La (**Figure 2B**), which are known to be highly associated with the disease (28, 29). In contrast, SLE samples displayed significantly higher numbers of reactivities but with a much smaller magnitude of induction (**Figure 2C**). The SLE group had anti-ssDNA, anti-riboprotein, and anti-histone antibodies in the Top 10 along with other nuclear targets.

**Figure 3:**
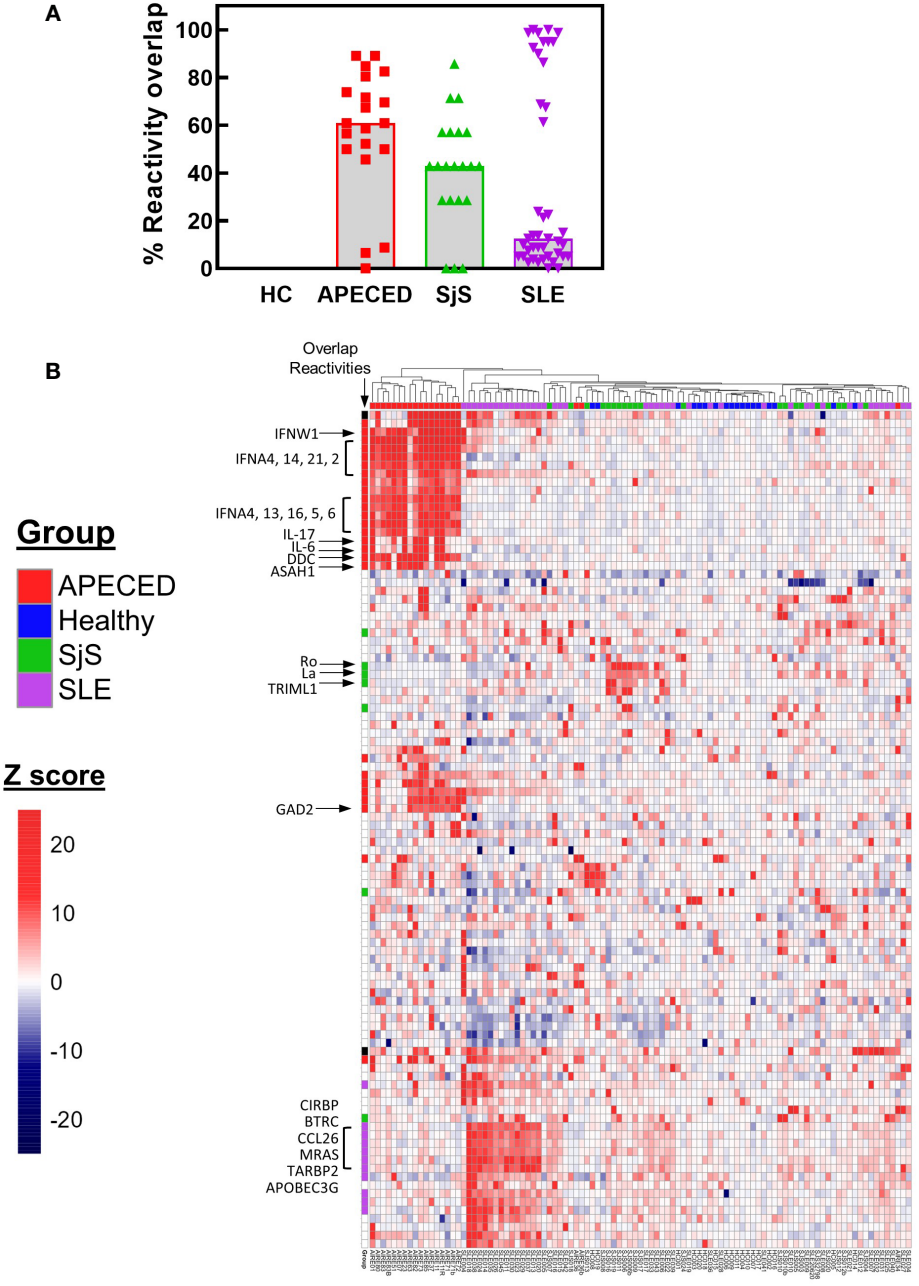
ProtoArray analysis of autoimmune patients reveals that autoantibody repertoires differ in the number of reactivities within and between groups. **(A)** The amount of overlap in the ProtoArray reactivity for each subject with other subjects in each group is shown. Overlap was considered for common reactivities with a Z >5 in 30% of the subjects for each group. **(B)** The highest 100 reactivities by variance are presented in a hierarchal clustered heat map. The heat map is colored by Z score and each column represents an individual subject with the group identified by the color bar above the column. Several key canonical reactivities are identified with arrows. Markings in the column labeled ‘Overlap’ identify which reactivities had a high degree of overlap for each group (Z >5 in 30% of the subjects for each group). APECED, autoimmune polyendocrinopathy–candidiasis–ecto-dermal dystrophy; HC, healthy control; SLE, systemic lupus erythematosus; SjS, Sjogren’s syndrome.

The ProtoArray data was then used to determine the number of common overlapping autoreactivities shared between subjects in each disease group. Reactivities with a Z score >5 and present in ≥30% of the subjects for each group were considered to be overlapping within that group. Based on these criteria there were 44 common overlapping reactivities for APECED, 7 for SjS, and 57 for SLE. Next, a value was calculated for each subject that showed their participation in their disease group’s overlap. To calculate this value, the number of reactivities with a Z score >5 that fell within the group’s overlap was tallied for each subject, and this number was divided by the total number of common reactivities present in that disease group (i.e., % reactivity overlap). A graph of the data (**Figure 3A**) showed a high degree of overlap for the majority of patients within the APECED and SjS groups, but the SLE group showed a bimodal distribution. To identify patterns of common reactivity present within and between disease groups, a heat map was constructed (**Figure 3B**) for the 100 highest variance reactivities across all groups. Hierarchical clustering of the data revealed disease-specific patterns. Patients with APECED have abundant anti-cytokine antibodies; those with SjS have strong reactivity for Ro-52 and La with virtually no additional autoreactivity. Patients with SLE cluster into two major groups, with differing levels of autoreactivity; one group with high reactivity against many shared proteins, and the second group with only weak-to-moderate reactivity that showed few shared reactivities. We termed the high reactivity SLE patients as ‘hot’ and the weaker reactivity patients as ‘cold’ and performed further analyses to try and understand their underlying differences.

**Figure 4:**
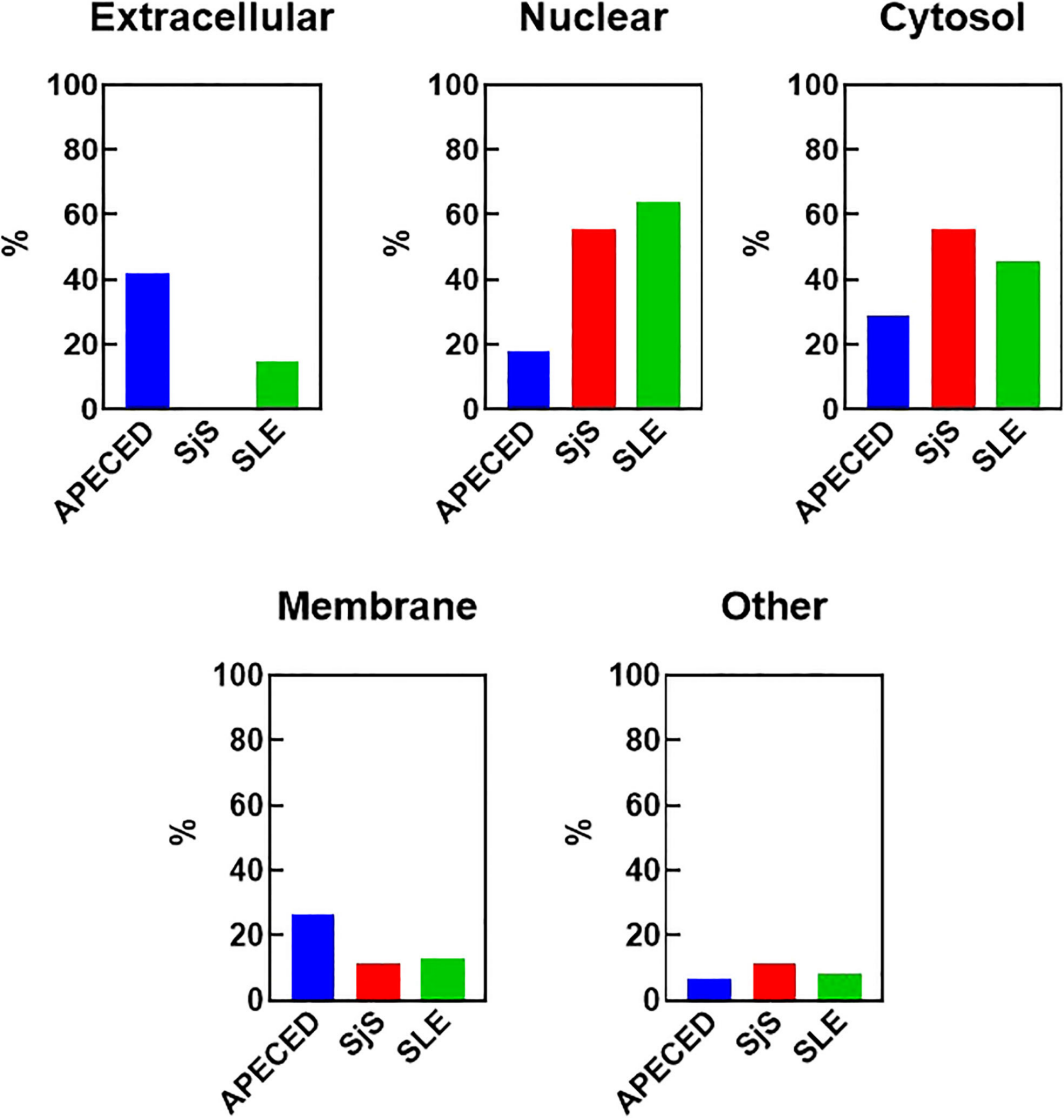
Cellular localization of the reactivities defining the different disease groups. The reactivities changed with a Z-score greater than 5 in at least 30% of the patients for each group were considered to be representative for each disease group. The cellular localization of the different antigens was determined using GO terms and also by manual curation for undefined genes. The percent of the group defining reactivities assigned to each cellular location are graphed. Some antigens have an unknown localization and were classified as “other”. An antigen may have more than one localization. APECED, autoimmune polyendocrinopathy–candidiasis–ecto-dermal dystrophy; SLE, systemic lupus erythematosus; SjS, Sjogren’s syndrome; GO, gene ontology.

One reason that there may be differences in the autoreactivity profiles of various diseases may be because of differences in antigen availability. To test this hypothesis, the antigens targeted by the common overlapping autoreactivities within each disease group were identified and classified based on their cellular localization (**Figure 4**). Notably, differences were found between APECED and SLE/SjS in the proportion of reactivities that were extracellular. APECED samples had a larger amount of reactivity against antigens located extracellularly (42.2% of total), which reflects the large number of anti-cytokine antibodies. In contrast, patients with SjS and SLE had a much higher percentage of autoreactivities against nuclear (55.6% and 63.9%, respectively) or cytosolic (55.6% and 45.9%, respectively) antigens.

### RNA-seq gene expression analysis

**Figure 5:**
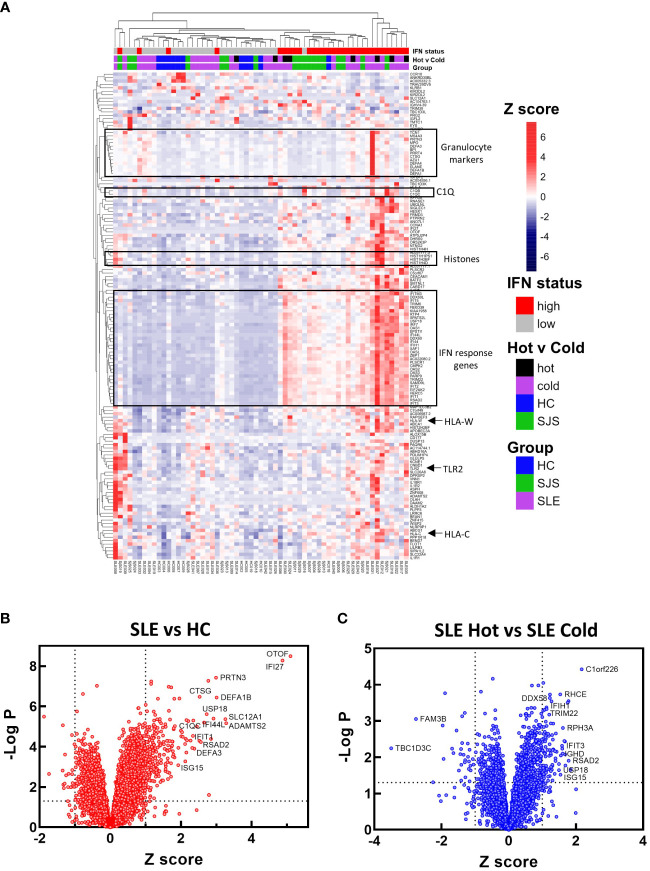
RNA-seq gene expression analysis. RNA-seq gene expression analysis was performed on blood samples from HCs, SjS, and SLE subjects. **(A)** Gene expression data is presented in a heat map as normalized expression to show comparisons for SLE versus HC or SLE ‘cold’ versus SLE ‘hot’. **(B, C) **Volcano plots were constructed for differential expression comparisons and dotted lines show a Z score cutoff of 0.7 and a p value cutoff of 0.01 and notable gene changes are labeled. HCs, healthy controls; SLE, systemic lupus erythematosus; SjS, Sjogren’s syndrome.

**Figure 6:**
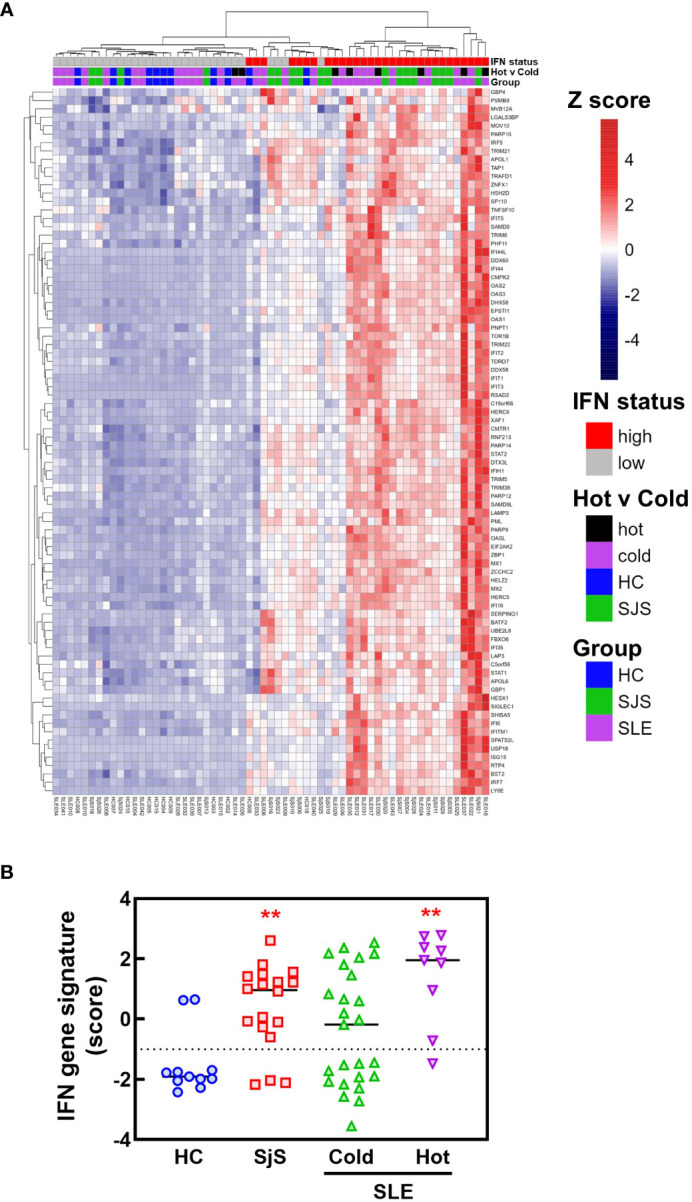
RNA-seq analysis of IFN response gene expression in SLE and SjS patients. RNA-seq analysis was performed on blood samples collected from HCs, SLE, and SjS subjects. **(A)** Z scores were calculated and a heat map was constructed to show the relative expression of 84 ImSig IFN pathway genes. **(B)** IFN gene signature scores were calculated using a panel of 9 IFN-regulated genes with the score for each subject represented by a different symbol and lines show the group medians. (**significantly different from HC ANOVA p<0.01). HCs, healthy controls; ImSig, immune cell type-specific gene expression signatures; SLE, systemic lupus erythematosus; SjS, Sjogren’s syndrome.

To identify mechanisms or pathways that contribute to the differences between the SLE ‘hot’ and ‘cold’ groups with either limited or broad autoreactivity, RNA-seq was performed on blood samples to identify differentially expressed genes (**Figure 5**). RNA-seq was also performed on peripheral blood from the HCs and the SjS patients to provide additional comparators for analysis. The results indicated that, as expected, there were several genes differentially expressed between SLE and HCs, with some of the same genes increased in SLE also increased in the SjS group. A volcano plot (**Figure 5B**) revealed that IFN-regulated genes (e.g. *IFI27* and *USP18*) and neutrophil markers (e.g. *CTSG* and defensins) were some of the notable differences between SLE and HCs. When comparing the ‘hot’ and ‘cold’ SLE groups (**Figure 5C**), there were notably IFN-regulated genes differentially expressed between the two groups (e.g. *DDX58, IFIH1* etc.). Additionally, when pathway analysis of ‘hot’ versus ‘cold’ was performed, several ‘hits’ related to IFN regulation were identified in the GO terms and Immune Signature (ImSig) database (**Table 1**). There was a robust increase in expression of a broad selection of IFN-regulated genes in the ‘hot’ patients (**Figure 6A**) and in SLE compared with SjS. As a more quantitative measure of IFN activation, a subset of highly validated IFN-response genes was selected as an IFN gene signature (GS), and scores were calculated based on these select genes. The IFN GS scores were generally higher in the ‘hot’ group, although some subjects in the SLE ‘cold’ group also had elevated IFN GS scores relative to HCs (**Figure 6B**). A typical bimodal IFN GS distribution was noted for SLE with nearly all the ‘hot’ group samples classified as IFN^hi^ compared to approximately 50% of the ‘cold’ group samples. A number of genes for B-cell activating factors or for total B-cell numbers were also investigated. However, no statistical differences were found between the groups (**Supplemental Figure 1**) suggesting that IFN activation of the cells or activation of the cells *via* increased BCR or TLR activation may be driving the ‘hot’ phenotype and it is less likely due to other B-cell activating factors or changes in total B-cell numbers.

**Table 1:** Pathway analysis of RNA-seq data.

Rank	Term	N Genes	Direction	P Value	FDR
1	lmSig_interferon	82	Up	1.11E-65	3.83E-61
2	GO_RESPONSE_TO_TYPE_I_INTERFERON	72	Up	1.83E-15	5.72E-13
3	GO_DEFESE_RESPONSE_TO_VIRUS	182	Up	2.01E-10	3.98E-08
4	GO_NEGATIVE_REGULATION_OF_VIRAL_GENOME_REPLICATION	44	Up	9.67E-10	1.75E-07
5	lmSig_neutrophiles	54	Up	2.44E-08	3.58E-06
6	GO_NEGATIVE_REGULATION_OF_VIRAL_PROCESS	71	Up	3.62E-08	5.13E-06
7	GO_RESPONSE_TO_VIRUS	242	Up	9.97E-08	1.32E-05
8	GO_REGULATION_OF_VIRAL_GENOME_REPLICATION	76	Up	2.91E-07	3.36E-05
9	GO_RESPONSE_TO_INTERFERON_ALPHA	18	Up	3.54E-07	4.01E-05
10	GO_INTERFERON_GAMMA_MEDIATED_SIGNALING_PATHWAY	75	Up	5.41E-07	5.96E-05

The genes differentially expressed for SLE ‘cold’ vs SLE ‘hot’ were subjected to pathway analysis and the top 10 GO terms and lmSig signatures that were significantly affected are shown.

### Next generation sequencing profiling of APECED, SjS, and SLE patient BCR repertoires

**Figure 7:**
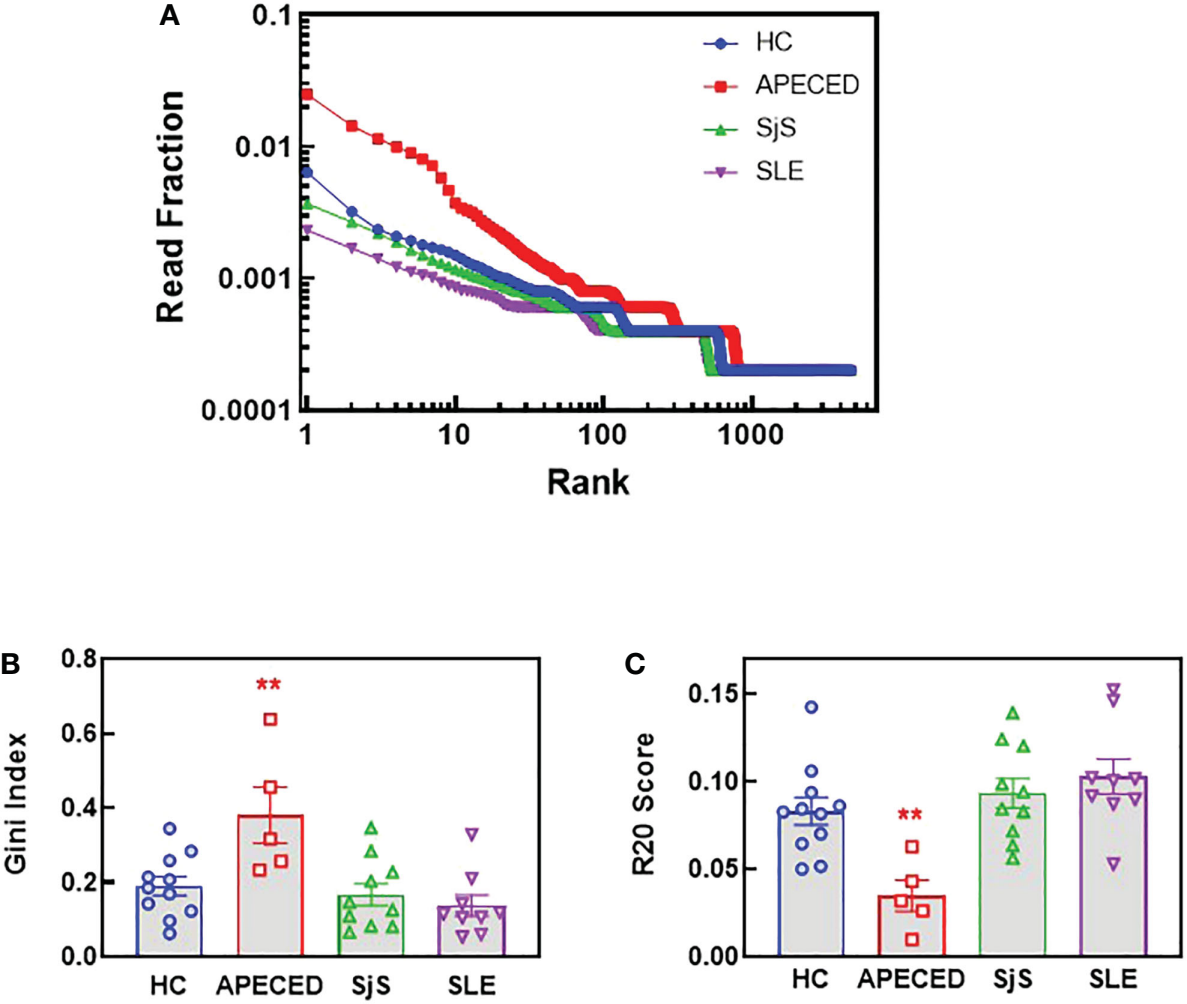
NGS BCR repertoire analysis of the sequence diversity and clonality of different disease groups. Switched memory B-cells were isolated from peripheral blood and RNA was purified for BCR sequencing. **(A)** The fraction of all reads for each clonotype were plotted with the clonotypes ranked from most abundant to least abundant for the top 4000 clonotypes. **(B)** The Gini score was calculated based on the clonotype frequency for each patient and the results are plotted by groups. **(C)** Clonality was determined for each subject and the R20 values for each subject are presented by group as the fraction of unique clones representing 20% of the sequenced repertoire, so the higher the R20 the less clonal dominance. (**significantly different from HC ANOVA p<0.01). APECED, autoimmune polyendocrinopathy–candidiasis–ecto-dermal dystrophy; BCR, B-cell receptor; HC, healthy control; NGS, next-generation sequencing; SLE, systemic lupus erythematosus; SjS, Sjogren’s syndrome.

Sequencing of BCRs was performed on switched memory B-cells isolated from the peripheral blood to gain further insight into the B-cell repertoires. For patients with APECED, the top ranked clones by read fraction were highly expanded and comprised a larger fraction of reads compared with the other groups (**Figure 7A**). In contrast, the SLE group had a greater fraction of reads represented by lower ranked clones, suggesting a broader repertoire, but few highly expanded clones. These data match the ProtoArray results indicating that patients with APECED had a more focused repertoire while those with SLE had a more broadly reactive, yet less expanded repertoire. As a quantitative measure of repertoire diversity, the Gini index (30) was calculated for each subject (**Figure 7B**). The Gini index was significantly higher for the APECED group compared with HCs and other autoimmune disease groups, indicating less diversity and greater expansion of a few clones. In contrast, the Gini index was slightly lower for the SjS and SLE groups compared with HCs, which likely reflects a more even distribution of clones and a broader autoimmunity. The R20 values (31) were also used to compare clonality between the groups (**Figure 7C**). For each subject, R20 values were presented by group as the fraction of unique clones representing 20% of the sequenced repertoire. The higher the R20 value, the less clonal dominance in the population. The R20 values were significantly lower for the APECED group indicating dominance of a few clones while there was a trend for slightly higher R20 values of SjS and SLE relative to HCs, suggesting a broader BCR repertoire.

**Figure 8:**
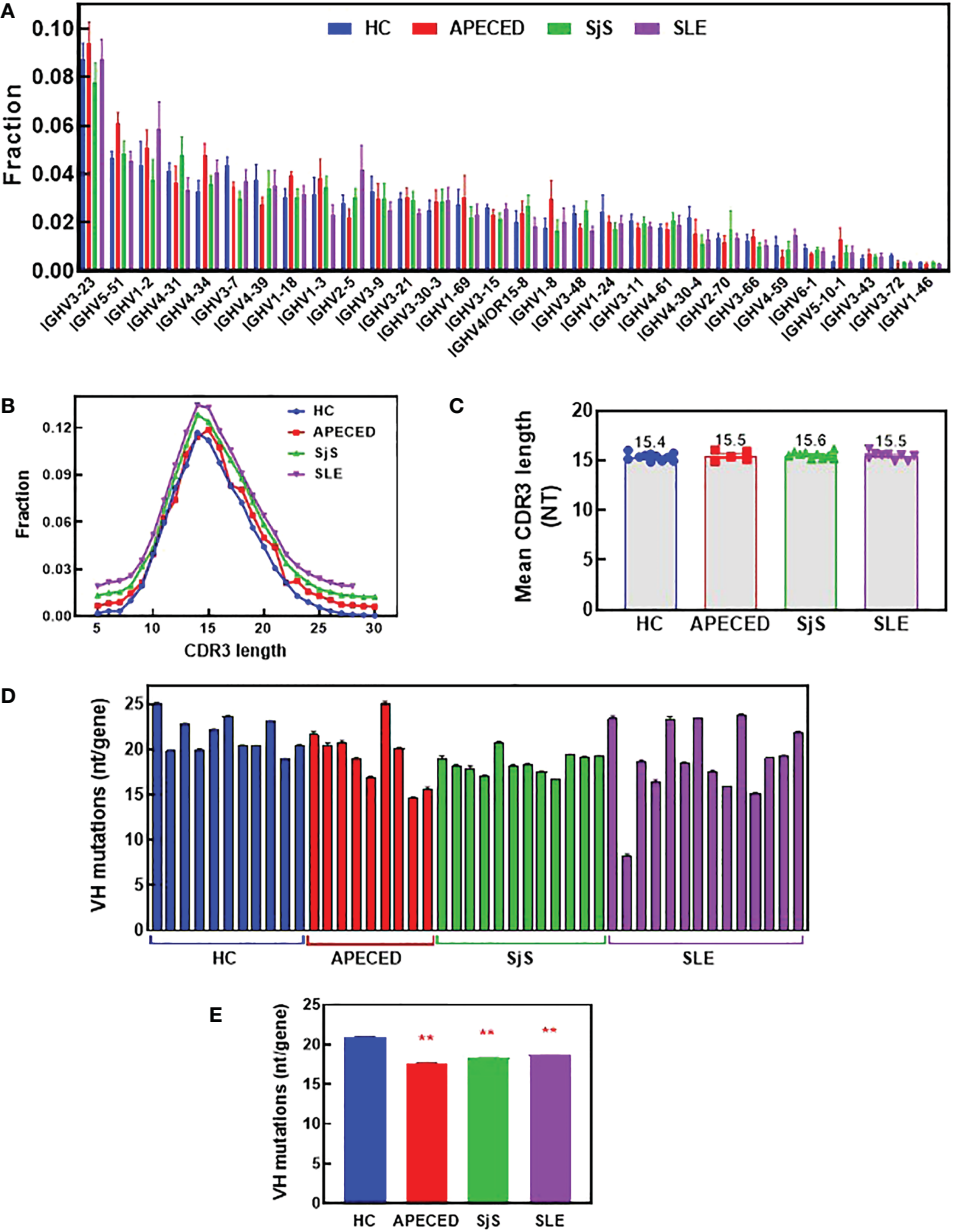
Analysis of VH gene family usage and CDR3 length. Switched memory B-cells were isolated from peripheral blood and RNA was purified for BCR sequencing. The relative frequency of VH gene sequences was determined and the mean was plotted for the top 30 most frequent VH genes for each group **(A)**. A Mann Whitney test with p value adjustment to account for multiple testing (FDR) was performed but no significant differences were found between groups for any of the genes. The CDR3 length distribution for all groups is plotted **(B)** and the mean CDR3 length for each subject was calculated and the group means are presented **(C)**. The number of mutations per VH gene was determined and the mean values are plotted for each individual **(D)** and the mean for the groups ± SEM **(E)**. A two-sided Mann Whitney U was run and found a significant difference between all the autoimmune groups and the HCs. (** significantly different from HC ANOVA p<0.01). APECED, autoimmune polyendocrinopathy–candidiasis–ecto-dermal dystrophy; BCR, B-cell receptor; CDR3, complementarity determining region 3; HC, healthy control; VH, immunoglobulin heavy chain variable region; SLE, systemic lupus erythematosus; SjS, Sjogren’s syndrome.

To try and better understand the processes causing the BCR repertoire differences, the immunoglobulin heavy chain variable region (VH) gene usage, complementarity determining region (CDR) 3 length, and VH mutation rate were investigated. **Figure 8A** shows VH family distribution as the fraction of all sequences. There have been reports suggesting there may be some changes in VH gene usage with autoimmunity (32–35), but we did not observe these previously reported differences. Next, average CDR3 length was determined and the distribution of CDR3 lengths (**Figure 8B**), along with the mean CDR3 length (**Figure 8C**) were plotted. CDR3 is one of the main determinants for antigen binding and longer CDR3s have been proposed to be prone to self-reactivity (36). However, we found no differences in CDR3 length for any of the patient groups compared with the HCs. The overall mutation rate and the rate in different VH domains was compared between the groups (**Figures 8D, E** and **Supplemental Figure 2**). We found there is a statistically lower somatic hypermutation (SHM) rate for all patients with autoimmune conditions versus HCs, and this difference has been noted previously in patients with SLE, SjS and RA (9, 37, 38). The main differences were found in the CDR1, CDR2, and Framework region 2, but the differences were global across all regions (**Supplemental Figure 2**).

## Discussion

The etiology of many autoimmune conditions is poorly understood but, in general, genetics and environmental exposures are the common underlying causes. An understanding of the pathogenesis of autoimmunity is often lacking and this is problematic as an improved understanding may better inform therapeutic treatment approaches. In this report, we have used two different approaches to characterize the B-cell repertoires of several different autoimmune diseases with a strong autoantibody component to try and learn more about the pathogenesis of autoimmunity. Our studies build on previous reports by using two complementary approaches (i.e., autoantibody profiling and BCR sequencing) to create a very detailed characterization of antibody diversity both at the protein and the transcript level.

While the ProtoArray technology used in this study does not allow quantitative statements about titers and affinities, it provides a comparative qualitative snapshot of autoantibody reactivities across three dissimilar autoimmune diseases. The ProtoArray is an array of 9,000+ antigens that provides semi-quantitative data on reactivity against a very wide range of antigens. The platform has been used extensively and validated and the strength of this platform is that it can be used to broadly characterize the IgG repertoires of diverse autoimmune patients in a holistic semi-quantitative manner. Although the ProtoArray does contain a large number of antigens, it does not contain all disease relevant ones such as 21-OH and NALP5 which are commonly seen in APECED patients. The weakness of this platform is that it is not as quantitative as other approaches such as ELISA. As the goal of our work was to perform broad profiling and repertoire comparison and not investigate individual reactivities quantitatively, the ProtoArray was the best choice. However, the presence of classical, disease-specific reactivities corroborates previous findings and validates the methodology used in this study, which allows us to translate this qualitative analysis to a more global perspective of autoantibody specificity distribution across SLE, SjS and APECED.

Our studies build on previous studies characterizing B cell autoimmunity in that we have compared a range of human autoimmune diseases that allows us to correlate antibody diversity to differing clinical manifestations, hypothesized disease mechanisms and breaks in tolerance. Overall, our autoantibody repertoire characterization provides insight into the different mechanisms of autoimmunity development in three diseases, accounting for differences in the autoantibody breadth, depth and antigen localization between the repertoires.

Other studies have also used ProtoArray profiling to characterize APECED and SLE autoantibody repertoires. Our data are generally consistent with other studies in terms of the number of reactivities that are elevated in the diseases relative to the HCs and in the identity of the elevated reactivities (12–14). However, our analyses also provide results characterizing the B-cell repertoires in a way that allows for a greater mechanistic understanding. We learned that APECED patients have a very focused repertoire of autoantibodies that are high affinity, as described previously (14), or are at high concentrations, which is in contrast to SLE patients that have a very broad repertoire, which is either of low affinity or at low concentrations. The SjS patients’ repertoire is different still with only two reactivities significantly elevated (Ro-52 and La) and a few others at low levels. There may be several mechanisms underlying these differences relating to breaks in tolerance in these diseases; for instance, the broad autoantibody repertoire in SLE patients suggests either weak polyclonal activation or broad cross-reactivity of a few clones. Although there are probably different mechanisms for loss of tolerance between the diseases, we can’t discount age at which autoimmunity develops and the age of the B cells as a factor as the median age of the APECED cohort is 30 and the SJS/SLE cohort is 56. Age-associated B cells (ABCs), which are in part TLR7-polyclonal activated, are prominent in lupus and there is decreased thymic function with age.

One possible difference between the examined diseases is the greater T-dependent B-cell activation that may occur in APECED patients (1, 39) versus the T-independent B-cell activation of SLE (23). T-cells that escape negative selection still need an activation trigger, which is not well understood in APECED. Once activated, the help provided to B-cells by T-cells is antigen-specific and restricted. This can result in greater affinity maturation and more focused clonal expansion. Indeed, clonal expansion was confirmed in the NGS BCR repertoire analysis. Interestingly, the rate of SHM is lower in all three autoimmune diseases relative to HCs. This suggests that extrafollicular B-cell activation could be occurring in all three autoimmune diseases, although it is probably a different mechanism between the diseases with T-cell help occurring in APECED and possibly TLR stimulation or other non-BCR activation signals driving the SjS/SLE patient B-cell activation. Alternatively, the abundance of self-reactive T-cells in APECED could lower the threshold for positive selection in germinal centers as limited T-cell help is generally thought to play a central role in selection and differentiation of high affinity B-cells (40). Additional cytokine stimulation may also be important for the development of autoimmunity. The observation that SLE and SjS showed high levels of IFN activity and the SLE ‘hot’ patients had broader autoreactivity and the highest IFN activity, suggest that direct and indirect IFN stimulation of B-cells may also contribute to their activation and production of autoantibodies. Previous reports have suggested that higher IFN and broader autoreactivity are linked (41–43) but our results show this correlation in very pronounced terms.

Another significant factor that may explain the differences in B-cell repertoire could be antigen availability. We found that the majority of the APECED patient autoantibodies are against extracellular antigens, while the very diverse repertoire of SLE patients is against nuclear or cytoplasmic antigens and less against extracellular antigens (12). These differences in antigen localization may be a function of disease pathogenesis. For example, in SLE there are large amounts of cell death and apoptosis resulting in the release of intracellular apoptotic or necrotic nuclear debris, which is coupled to defects in the clearance of these potentially immunogenic autoantigens that can result in broad autoreactivity (44). The nuclear and cytoplasmic proteins and nucleic acids released upon dysregulated cell death in SLE may become antigenic and activate B-cells, giving rise to antibodies targeting these self-antigens. Furthermore, the nuclear antigens in SLE can lead to peripheral polyclonal B-cell activation through nucleic acid sensors such as TLR7 and TLR9, which can lead to breaks in peripheral tolerance and may contribute to the very broad reactivity profile in the disease (22, 45). Studies showing TLR7-dependent activation of extrafollicular B-cells in SLE support this hypothesis (23). Why SjS patients have reactivity focused on only Ro-52/La is unclear. It is possible for SjS that TLR activation of B-cells plays a role in development of autoimmunity, but possibly only a limited availability of antigens results in a less broad autoantibody repertoire, which is also reflected in the more anatomically focused pathology.

Our studies focused on sequencing BCRs from switched memory B-cells as these were the cells available in the peripheral blood most likely to reflect the more persistent autoantibody repertoire. However, it would also be interesting to sequence BCRs from plasmablasts in secondary lymphoid organs and plasma cells residing in the bone marrow as they also contribute to autoimmunity and best reflect the reactivities of circulating autoantibodies. The population of cells studied may be important as another study reported both increased clonality and diversity of unswitched B-cells with SLE (21), which contrasts with our findings in switched memory B-cells. However, the report did also attribute the B-cell activation in SLE to a microbial source that may be *via* TLR stimulation. Similarly, rare bone marrow plasma cells in APECED may reveal the expected higher SHM rates as indicated by affinity measurements of APECED autoantibodies (14). Nonetheless, using our ProtoArray and NGS results, it can be concluded that patients with APECED have more clonal expansion and less repertoire diversity compared with HCs, while patients with SLE have a broader autoreactive B-cell population that is less clonally expanded. Future studies matching the IgG reactivity produced by a single B-cell with the BCR sequence would also be interesting as it could allow for an even more detailed picture of B-cell repertoires and would allow for a focus on the clones producing the autoreactive pathogenic autoantibodies.

**Figure 9:**
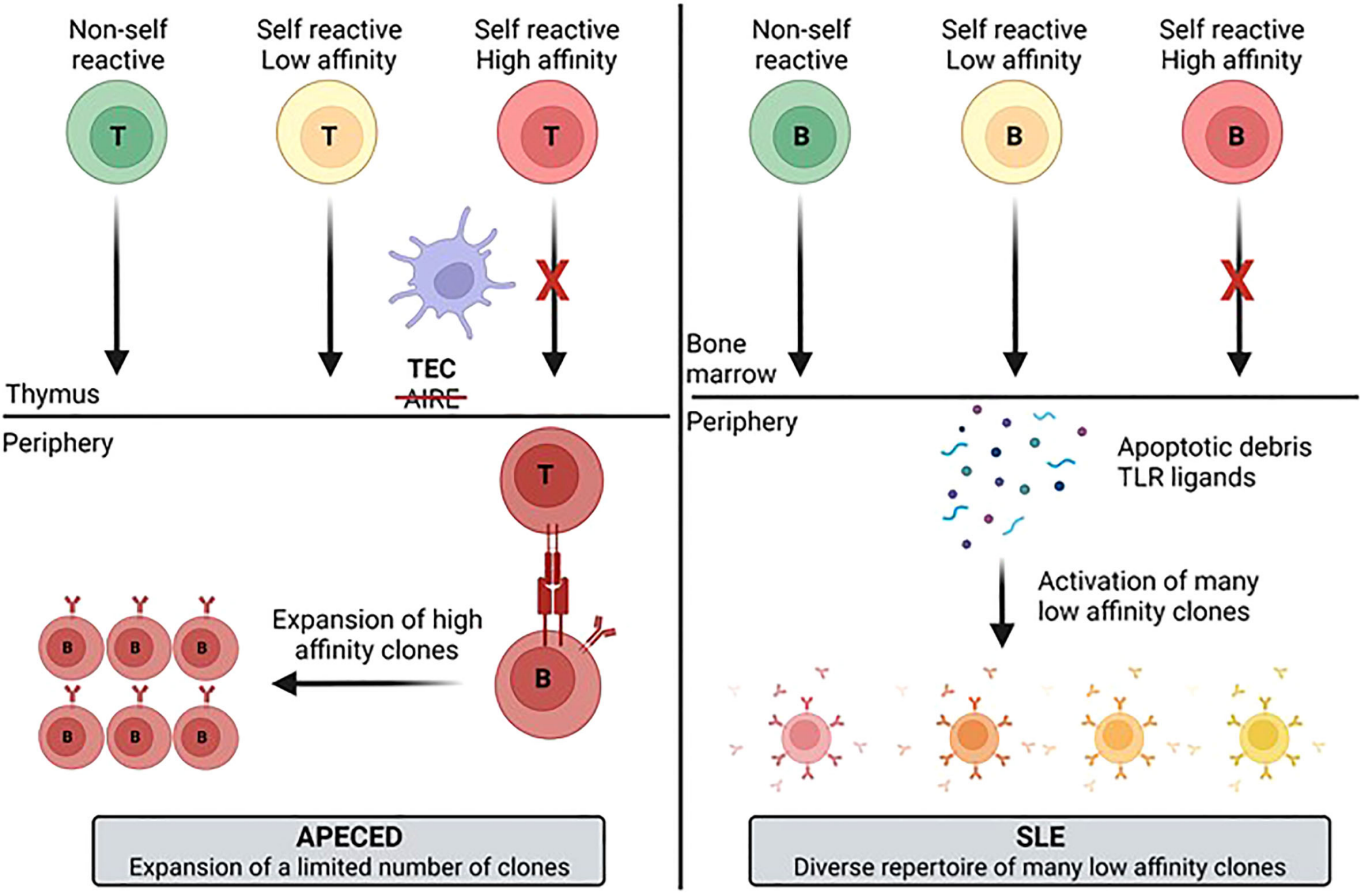
Model for development of the autoimmune B-cell repertoire for different diseases. The differences in the diversity and clonotype expansion of the autoimmune repertoire of different diseases may be a function of multiple variables including antigen availability, the presence of PRR ligands that activate B-cells, and T-cell help. These different factors likely contribute to generation of a more focused and clonally expanded repertoire in APECED patients and a more diverse, but less expanded repertoire in SLE patients. APECED, autoimmune polyendocrinopathy–candidiasis–ecto-dermal dystrophy; PRR, pattern recognition receptor; SLE, systemic lupus erythematosus.

Based on our results we propose a model (**Figure 9**) to explain the autoimmune mechanisms underlying APECED and SLE. Understanding how pathogenic B-cells arise may be useful for selecting new treatment approaches. Inhibition of B-cells has proven to be effective for treatment of SLE as evidenced by the approval of belimumab and the efficacy seen for the BAFF/APRIL inhibitor, atacicept. It will be interesting to see if TLR7/8 inhibitors (enpatoran, afimetoran and others) provide efficacy in SLE through inhibition of B-cell activation, reduction in IFN activity, or both as anifrolumab (anti-IFN receptor) has been found to be efficacious, but its impact on B-cells has not been well characterized (46). Uncovering additional targets that contribute to B-cell activation in SLE, or other similar polygenic autoimmune diseases (such as SjS), may result in development of other efficacious therapies. No specific treatment exists for APECED but combined T-cell or B-cell targeting has shown promise for autoimmune pneumonitis (47). Targeted inhibition of autoreactive T-cells or T-cell help to B-cells could be a goal of treatment. Hopefully, the work presented here may contribute to the development of new treatment options for these different patient groups.

## Data availability statement

The RNA-seq gene expression data has been uploaded to NCBI GEO under the accession number GSE222408 and ProtoArray autoantibody profiling results can be made available upon request.

## Ethics statement

The studies involving human participants were reviewed and approved by the institutional review boards of the Mayo Clinic and the National Institute of Allergy and Infectious Diseases (NIAID). Study participants provided written informed consent to participate in the study, and sampling was conducted using protocols approved by the Mayo Clinic Institutional Review Board and in accordance with the Declaration of Helsinki. For details of the APECED patient study see clinicaltrials.gov listing NCT01386437. The patients/participants provided their written informed consent to participate in this study.

## Author contributions

SK, SO, MS, QA MJ, TN, ML, JV, JD, AB contributed to the research design of the studies. The experimental execution was carried out by TC, PD, SO, JZ, ET, MJ, EF, and AB. Data analysis was performed by TC, PD, SK, SO, MS, QA, ET, MJ, TN, EF, JN, ML, JV, and AB. Preparation of the manuscript was performed by TC, PD, SK, SO, MJ, TN, ML, and AB. All authors contributed to the article and approved the submitted version.
