# Supplementary material for: Autoantibody repertoire characterization provides insight into the pathogenesis of monogenic and polygenic autoimmune diseases
Source: Front Immunol. 2023 Feb 10;14:1106537. doi: 10.3389/fimmu.2023.1106537 (PMC9955420; doi:10.3389/fimmu.2023.1106537)

Supplemental Figure 1. Markers of B cell activation and expansion

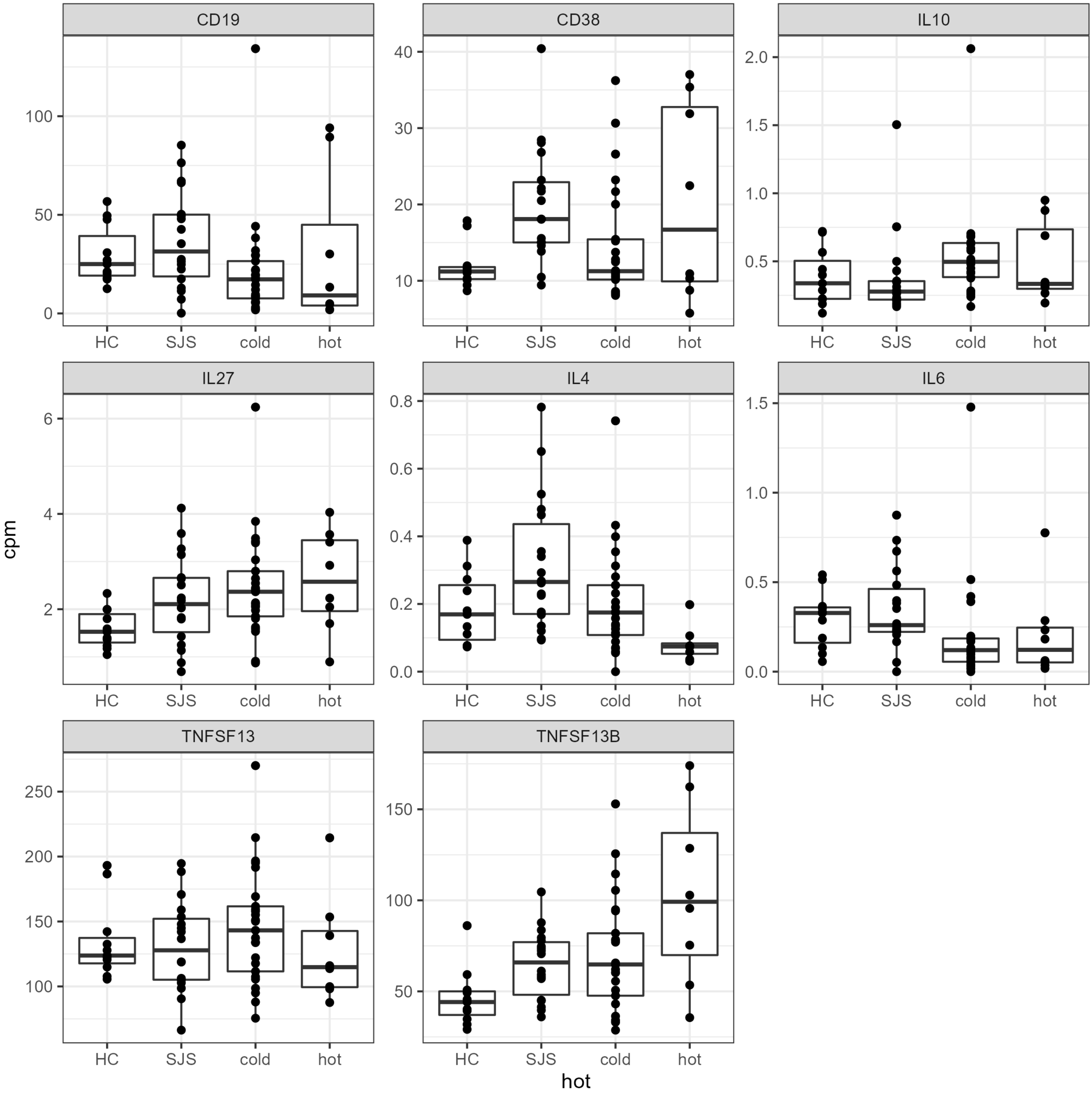

Supplemental Figure 2. Mutations detected per VH gene domain. The number of mutations for each region of the VH gene were determined. Graphs show the mean + SEM for all the subjects in each group.

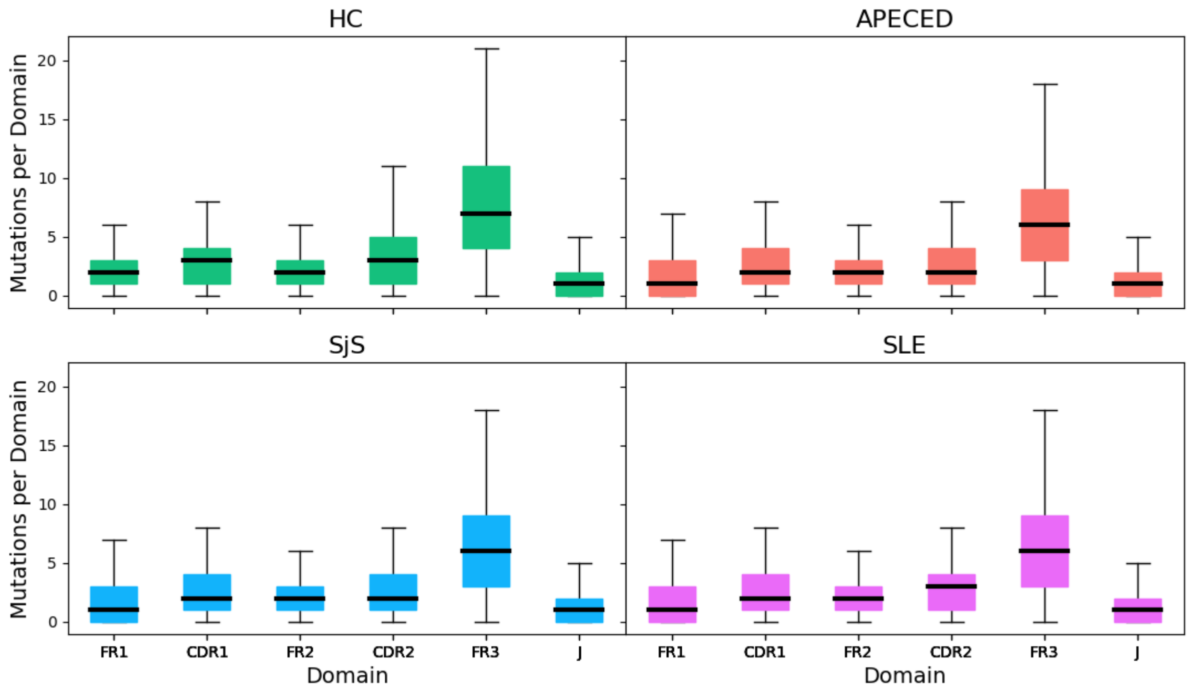

Supplemental Figure 3. Memory B cell isolation. Switched memory B cells were isolated using Miltenyi MACS beads and flow cytometry was used to confirm their purity.

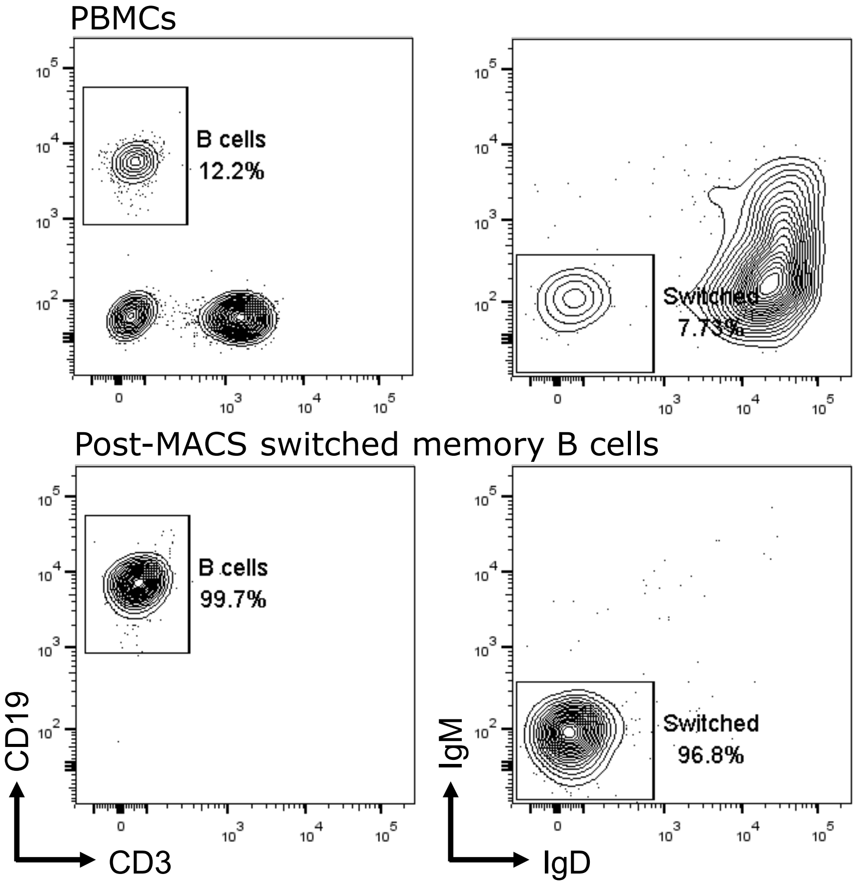

Supplement: Supplementary file 1 [file DataSheet_1.pdf]
